# Supplementary material for: Plain language summaries: A systematic review of theory, guidelines and empirical research
Source: PLoS One. 2022 Jun 6;17(6):e0268789. doi: 10.1371/journal.pone.0268789 (PMC9170105; doi:10.1371/journal.pone.0268789)
Supplement: S1 Table — (PDF) [file pone.0268789.s001.pdf]

S 1 Table. Studies included in this review.

Authors	Year	Study Type
Alderdice et al.	2016	Empirical, quantitative: PLS vs PLS
Anstey	2014	Theoretical, other
Anzinger et al.	2020	Empirical, quantitative: PLS vs other
American Psychological Association	2018	Guideline
Barbara et al.	2016	Empirical, qualitative: PLS vs other
Barnes & Patrick	2019	Theoretical, review
Barnfield et al.	2017	Empirical, evaluative
Bredbenner & Simon	2019	Empirical, quantitative: PLS vs other
Brehaut et al.	2011	Empirical, evaluative
Brown et al.	2012	Theoretical, other
Buljan et al.	2018	Empirical, quantitative: PLS vs other
Buljan et al. (BMC)	2020	Empirical, quantitative: PLS vs PLS
Buljan et al. (Trials)	2020	Empirical, evaluative
Busert et al.	2018	Empirical, evaluative
Carvalho et al.	2019	Theoretical, review
Cerejo et al.	2021	Empirical, evaluative
Cochrane	2020	Theoretical, other
Cochrane Methods	2013	Guideline
Cochrane Norway	2019	Guideline
Cochrane Norway	2017	Guideline
Coomarasamy et al.	2001	Theoretical, other

Corneli et al.	2020	Empirical, evaluative
Dear et al.	2011	Theoretical, other
Dormer & Walker	2020	Theoretical, other
Dubé & Lapane	2014	Guideline
Duke	2015	Theoretical, other
Duke	2012	Guideline
Ellen et al.	2014	Empirical, qualitative: PLS vs PLS
Expert group on clinical trials for the implementation of Regulation (EU) No 536/2014	2017	Guideline
Ferrar & Conran	2021	Empirical, quantitative: PLS vs other
FitzGibbon et al.	2020	Theoretical, review
Glenton	2017	Empirical, evaluative
Glenton et al.	2010	Empirical, qualitative: PLS vs PLS
Green-Brown & Wigington	2012	Theoretical, other
Gudi et al.	2021	Theoretical, other
Halprin	2021	Guideline
Hauck	2019	Theoretical, other
Jakus et al.	2021	Empirical, evaluative
Jelicic Kadic et al.	2016	Theoretical, review
Karačić et al.	2019	Empirical, quantitative: PLS vs other
Kaslow	2015	Theoretical, other
Kerwer et al.	2021	Empirical, quantitative: PLS vs PLS
King et al.	2017	Theoretical, other
Kirkpatrick et al.	2017	Empirical, quantitative: PLS vs PLS
Koufogiannakis et al.	2016	Theoretical, other

Kuehn	2017	Theoretical, other
Kuehne & Olden	2015	Theoretical, other
Langendam et al.	2013	Theoretical, other
Linte	2009	Theoretical, other
Lionbridge	2019	Guideline
Lobban et al.	2021	Guidance-related
Lugaz	2021	Theoretical, other
Maguire & Clarke	2014	Empirical, quantitative: PLS vs other
Manighetti et al.	2021	Theoretical, other
Maurer et al.	2021	Empirical, evaluative
Mellalieu	2018	Theoretical, other
NIHR	2021	Guideline
Nunn & Pinfield	2014	Empirical, evaluative
Opiyo et al.	2013	Empirical, quantitative: PLS vs other
Penlington et al.	2020	Empirical, evaluative
Phung et al.	2020	Theoretical, other
Pushparajah et al.	2018	Empirical, evaluative
Rader et al.	2014	Theoretical, review
Rakedzon et al.	2017	Empirical, quantitative: PLS vs other
Raynor et al.	2018	Empirical, evaluative
Rees et al.	2017	Guidance-related
Richter	2008	Theoretical, other
Rodgers	2017	Theoretical, other
Rosenbaum	2010	Theoretical, other

Santesso et al.	2008	Theoretical, other
Santesso et al.	2015	Empirical, quantitative: PLS vs PLS
Seidel et al.	2017	Theoretical, other
Shailes	2017	Theoretical, review
Silvagnoli et al.	2020	Empirical, quantitative: PLS vs PLS
Simmons	2012	Theoretical, other
Stricker et al.	2020	Empirical, quantitative: PLS vs other
Suart et al.	2020	Empirical, evaluative
Taylor et al.	2018	Theoretical, review
The Steering Group of the Campbell Collaboration	2016	Guideline
TransCelerate Biopharma Inc.	2015	Guideline
Wada et al.	2020	Theoretical, other
Walker & Dormer	2021	Guidance-related
Whiting et al.	2018	Guidance-related
Zhang et al.	2020	Theoretical
eLife: PLS: Results of reader survey	2017	Empirical, evaluative
eLife: PLS: How to write an eLife digest	2017	Guideline
“AGU” Tips for Plain Language Summaries	2020	Guideline
“Autism” Manuscript Submission Guidelines	2020	Guideline
“People and Nature” PLS Guidelines	2020	Guideline
“Taylor & Francis” PLS Guidelines	2021	Guideline
