# Supplementary material for: Plain language summaries: A systematic review of theory, guidelines and empirical research
Source: PLoS One. 2022 Jun 6;17(6):e0268789. doi: 10.1371/journal.pone.0268789 (PMC9170105; doi:10.1371/journal.pone.0268789)
Supplement: S2 Table — (PDF) [file pone.0268789.s002.pdf]

**S2 Table. PLS guidelines and criteria.**

| Guideline | Linguistic Attributes | Formal Attributes | General Content | Presentation of Results | Presentation of Quality of Evidence | Contextual Information |
|---|---|---|---|---|---|---|
| **American Psychological Association** (“translational abstract”), psychological science | Tone:<br>▪ more personal and friendly than scientific abstract<br>▪ do not overstate / oversimplify findings or conclusions<br>Words: -<br>Sentences: - | Text Length:<br>▪ 150 - 200 words<br>Text Structure: -<br>Use of Tables/Visuals: - | Title: -<br>Content structure:<br>▪ follow a pattern of introduction, method, results, discussion<br>Headlines: -<br>Content:<br>▪ consider your audience<br>▪ clearly describe investigated problem<br>▪ describe participants only insofar as relevant to the audience; details of the sample only if it is remarkable in some way<br>▪ detailed information about study methods may be summarized or omitted<br>▪ emphasize conclusions that are relevant for the audience<br>▪ try to create a “take home message” | ▪ state the findings in clear, nontechnical language<br>▪ remove any statistics<br>▪ if article contains more multiple studies, summarize contents of all studies | - | Purpose of text:<br>▪ clear communication of article content<br>▪ emphasis on article’s value to educated public/professional audiences<br>Author of PLS: -<br>Review of PLS: -<br>Access: -<br>Context:<br>▪ recommendation for PLS as standard feature of each APA journal article |
| **Cochrane 2013, standards for the reporting of PLS** (“Plain Language Summaries”), Cochrane Intervention Reviews | Tone: -<br>Words:<br>▪ avoid technical terms and jargon (explain them if unavoidable)<br>▪ avoid long words / words with many syllables / misunderstandable words<br>▪ consider introducing an acronym or short term for repeated use<br>▪ avoid regional terms | Text Length:<br>▪ 250 - 300 words<br>Text Structure:<br>▪ use short paragraphs<br>Use of Tables/Visuals: - | Title:<br>▪ restate review title in plain language if possible, otherwise explain terms<br>▪ avoid recommendations<br>Content structure:<br>▪ review question<br>▪ background<br>▪ study characteristics<br>▪ key results<br>▪ quality of evidence<br>Headlines: | ▪ present results for all main outcomes<br>▪ use consistent wording across outcomes<br>▪ report findings on harms that are described in the review and state whether they have been fully reported in the included studies<br>▪ it is not essential to provide numerical data<br>▪ do not present | ▪ describe overall quality of evidence for each of the main outcomes based on GRADE considerations<br>▪ describe any factor that could affect the confidence in the results<br>▪ provide key reasons for quality of evidence / limitations in lay terms<br>▪ describe if quality of evidence is high | Purpose of text:<br>▪ summary of the review that contains the crucial information in plain language and that will be understood by the general public<br>Author of PLS: -<br>Review of PLS: -<br>Access: -<br>Context:<br>▪ tailor messages across different summary versions of the review |

| | | | | | | |
|---|---|---|---|---|---|---|
| | (AE / BE)<br>▪ use active voice<br>Sentences:<br>▪ one keypoint / sentence<br>▪ avoid more than two hard words in a sentence unless you can explain them | | ▪ standard headings<br>▪ consistent order<br>▪ in bold type<br>Content:<br>▪ convey the question addressed in the review<br>▪ shortly describe population, intervention and outcomes in Background section<br>▪ give enough detail on study characteristics (incl. search date and population details) | numerical data if estimations of effects are imprecise or uncertain<br>▪ if numerical data is provided, use natural frequencies for dichotomous outcomes and accompany relative effects with absolute effect estimates<br>▪ explain any statistical term<br>▪ statics, if used, should provide valid, digestible summary of direction, size and precision of the effect estimates | ▪ if impact of funding sources on quality of the evidence is considered in the review, include a statement in the PLS | ▪ consistent reporting of key messages between PLS, main text, SoF table and authors' conclusions. |
| **Cochrane 2016, Checklist for PLS Review** ("Plain Language Summaries"), Cochrane Intervention Reviews | Tone: -<br>Words:<br>▪ avoid or explain acronyms, jargon and technical terms<br>▪ PLS authors can choose to refer to "we" or to "review authors", but be consistent<br>Sentences: - | Text Length:<br>▪ 400-700 words<br>Text Structure: -<br>Use of Tables/Visuals: - | Title:<br>▪ if title is difficult to understand, consider re-writing it in plain language<br>Content structure:<br>▪ What is the aim of review?<br>▪ Key messages<br>▪ What was studied in the review?<br>▪ What are the main results of the review?<br>▪ How up-to-date is this review?<br>Headlines:<br>▪ use sub-headings<br>Content:<br>▪ explain that results come from a systematic study rather than a single study<br>▪ describe if necessary: why this topic is important; the population; the | ▪ present results only for the most important outcomes, try not to present more than 7 outcomes<br>▪ if no data was found, present outcomes nevertheless<br>▪ present results consistently (similar words / expressions for similar effects)<br>▪ use the provided standard sentences<br>▪ use absolute numbers (not relative risks, odds ratios, or percentages) | ▪ present quality or certainty for each outcome as presented in the SoF table<br>▪ if quality/certainty is not high, avoid strong statements, add modifying statements | Purpose of text: -<br>Author of PLS: -<br>Review of PLS:<br>▪ the checklist is intended for use by reviewers of PLS<br>Access: -<br>Context:<br>▪ a template, PLS examples and standardized statements are provided<br>▪ ensure results are reported consistently between PLS and main text / abstract / SoF table |

| | | | | | | |
|---|---|---|---|---|---|---|
| | | | intervention; the comparison group; the outcomes; possible adverse effects<br>▪ do not present recommendations | | | |
| **Cochrane 2019** [1]<br>("Plain Language Summaries"), Cochrane Intervention Reviews | Tone: -<br>Words:<br>▪ avoid research jargon<br>▪ refer to "study" rather than "trial"<br>▪ use name of the outcome and name of intervention instead of "outcome" / "intervention"<br>▪ use words like "parents", "women", etc. instead of "participants"<br>Sentences: - | Text Length:<br>▪ *see above*<br>Text Structure: -<br>Use of Tables/Visuals:<br>▪ if PLS is published outside Cochrane Library, add a simplified version of SoF table | Title:<br>▪ *see above*<br>Content structure:<br>▪ *see above*<br>Headlines:<br>▪ *see above*<br>Content:<br>▪ describe if necessary: why this topic is important; the population / health problem addressed in the review; the intervention and what it was compared to; the outcomes.<br>▪ explain that results come from a systematic study rather than a single study<br>▪ do not present recommendations | ▪ *see above*<br>▪ use standardized qualitative statements when reporting effects of an intervention<br>▪ ideally, use numbers and words; if you use numbers, present them in parentheses after the qualitative statement<br>▪ use absolute numbers (not relative risks, odds ratios, percentages or numbers needed to treat)<br>▪ when presenting continuous outcomes using numbers, refer to the scale | ▪ *see above*<br>▪ presenting confidence intervals is mostly not necessary but it may be useful in some situations (example statement is provided) | Purpose of text: -<br>Author of PLS: -<br>Review of PLS: -<br>Access: -<br>Context:<br>▪ *see above* |
| **The Steering Group of the Campbell Collaboration (2016)**<br>("Plain Language Summaries"), Campbell systematic reviews | Tone:<br>▪ use direct language<br>▪ accessible manner<br>Words:<br>▪ avoid jargon<br>▪ specific terms that are familiar to policy makers and practitioners should be retained<br>▪ verbs: present tense<br>Sentences: - | Text Length:<br>▪ 600 - 750 words<br>Text Structure: -<br>Use of Tables/Visuals:<br>▪ text box: "What is the aim of this review?"<br>Form:<br>▪ no footnotes or references | Title:<br>▪ headline style<br>▪ summarizing main findings<br>▪ reference to full title at the end of the PLS<br>Content structure:<br>▪ see "Headlines"<br>Headlines:<br>▪ use headings and additional subheadings if needed<br>▪ Headlines: The Review in Brief; What is this Review about?; What are the main findings of this | ▪ report the study findings directly and in present tense<br>▪ avoid selective outcome reporting<br>▪ use qualitative statements when presenting the effects of the intervention<br>▪ avoid numbers to increase accessibility<br>▪ qualitative statements: similar words and expressions for similar levels of effect (additional appendix with standardised | ▪ included studies: optionally add statement about the quality of evidence<br>▪ results: GRADE reporting system<br>▪ decide whether the size of the effect is important, less important or not important to the user<br>▪ use standardised statements about effect<br>▪ presenting confidence intervals is mostly not necessary but it may be useful in some | Purpose of text:<br>▪ to make information about main findings available in an easily understandable format<br>▪ to report this information in a consistent way<br>Author of PLS:<br>▪ study authors submit PLS with final review<br>Review of PLS:<br>▪ edited by the secretariat, revised version checked with lead study author<br>▪ meta-information |

| | | | | | | |
|---|---|---|---|---|---|---|
| | | | review?; What do the findings of this review mean?; How up-to-date is this review?; What is the Campbell Collaboration?; About this summary<br>Content:<br>▪ assessed interventions (i.a.)<br>▪ primary outcomes<br>▪ results for each outcome and analysis of heterogeneity<br>▪ discussion of theory of change (i.a.)<br>▪ quality of evidence<br>▪ implications for policy, practice, research (i.a.) | statements) | situations (standardized statement is provided) | reported as last sentence in PLS<br>Access: -<br>Context:<br>▪ results should be reported consistently between PLS and main text / abstract<br>▪ a template with suggested wording is provided |
| **Expert group** on clinical trials for the implementation of Regulation (EU) No 536/2014 ("lay summary" / "summary of clinical trial results for laypersons"), clinical trial results | Tone:<br>▪ avoid any promotional language<br>Words:<br>▪ avoid jargon / technical / medical / scientific language<br>▪ remove unnecessary / complex words<br>▪ be consistent with use of words and define them<br>▪ explain underlying concepts if necessary<br>▪ avoid ambiguous words / phrases<br>▪ limit use of acronyms, and of abstract or multisyllabic words<br>▪ verbs: use active rather than passive voice<br>▪ texts should be aimed at literacy proficiency level of 2-3 (use metrics to measure text readability)<br>Sentences: | Text Length:<br>▪ as short as possible<br>Text structure:<br>▪ bullet points<br>▪ white space between topics<br>▪ minimum of 12-point font<br>▪ avoid text in all caps or underlining<br>Use of Tables/Visuals:<br>▪ use visuals to convey critical concepts but limited use of unnecessary images<br>▪ visuals should present one message per image and be clearly labelled with captions | Title:<br>▪ specific to the trial<br>▪ may be shortened / simplified<br>Content structure:<br>▪ present general information before the details<br>Headlines:<br>▪ use headlines and descriptive subheadings to organize information<br>Content:<br>▪ focus on unambiguous, factual information<br>▪ no promotional content should be included<br>▪ avoid overwhelming with too much information<br>▪ describe adverse reactions | ▪ describe every study arm and outcome<br>▪ Numbers: follow numeracy principle (links to additional information is provided)<br>▪ report frequencies both in numerical terms and percentages<br>▪ report all outcomes | ▪ don't include promotional content | Purpose of text:<br>▪ EU Clinical Trials Regulation 536/2014 (Article 37): sponsors need to provide summary results in a format understandable to laypersons (research participants and general public)<br>Author of PLS: -<br>Review of PLS:<br>▪ consider including patients, patient representative or advocates to ensure the summary meets its aims; consider help of medical writers with plain language expertise<br>Access:<br>▪ made available in the EU Portal and Database<br>Context:<br>▪ provide summary as a |

| | | | | | | |
|---|---|---|---|---|---|---|
| | ▪ make short and succinct sentences instead of long and complex ones | | | | | minimum in local language of where the trial took place<br>▪ where possible include English version<br>▪ a template with 10 elements that should be included in the summary and suggested wording is provided |
| **Lionbridge (2019)**: Seven rules for effective communication (“Plain Language Summaries”), clinical trial results | Tone:<br>▪ information should be presented factually and neutrally<br>▪ respectful tone so that study participants do not feel as victims; avoid marketing jargon or overly positive presentation<br>Words:<br>▪ simple and unambiguous<br>▪ avoid polysyllabic words; complex technical terms (unless you explain them); scientific jargon that may be misunderstood or cause confusion<br>Sentences:<br>▪ active sentences with a subject that executes an action<br>▪ short, simple sentences<br>▪ avoid subclauses and nested sentences | Text Length: -<br>Form: -<br>Use of Tables/Visuals:<br>▪ use visuals aids such as empty spaces or graphs if it enhances communication with the target group | Title: -<br>Content structure: -<br>Headlines: -<br>Content:<br>▪ content and presentation should be oriented towards language, style and knowledge of laypersons | ▪ report absolute, whole numbers<br>▪ report numbers without decimal places or as percentages<br>▪ avoid probabilities or relative risks | - | Purpose of text:<br>▪ to inform persons with average education level about results and conclusions of clinical studies<br>Author of PLS: -<br>Review of PLS: -<br>Access:<br>▪ access to extensive guidelines for writing PLS only for members<br>Context: - |
| **TransCelerate Biopharma Inc.** (“lay summary”), clinical trial results | Tone:<br>▪ factual and objective tone<br>Words:<br>▪ avoid superlative and enthusiastic words<br>▪ use neutral language | Text Length: -<br>Form:<br>▪ use material that is fair and balanced in terms of formatting<br>Use of Tables/Visuals:<br>▪ do not use materials | Title: -<br>Content structure: -<br>Headlines: -<br>Content:<br>▪ factual and objective content<br>▪ use accurate, | ▪ make only comments on the outcome that are factual in nature<br>▪ do not make inferences or assessments<br>▪ link to additional neutral language guide | ▪ include a statement that results are from a single trial and different results may be obtained from other studies<br>▪ include a statement that | Purpose of text:<br>▪ written to be understandable to the general public<br>▪ recommendations to help prepare PLS that are not perceived as |

| | | | | | | |
|---|---|---|---|---|---|---|
| | (link to additional guidance is provided)<br>Sentences: - | that have a commercial appearance<br>▪ do not use brand colors / logos<br>▪ be fair and balanced in terms of formatting | non-misleading information<br>▪ include information on efficacy and safety data from the trial<br>▪ do not provide approval status<br>▪ material should be fair and balanced | is provided | no therapeutic changes should be made based on the results of a single trial without consulting a healthcare professional | promotional<br>Author of PLS:<br>▪ sponsor of trial<br>Review of PLS: -<br>Access:<br>▪ posted to public websites, EU database<br>▪ provided to trial participants<br>Context:<br>▪ take care that PLS is only provided in a non-promotional context<br>▪ add a statement with link to ClinicalTrials.gov & EU clinical trial register |
| **Duke (2012)**: How to write a lay summary ("lay summary"), Digital Curation Center collaborating with Patients Participate! project | Tone:<br>▪ do not write to entertain<br>Words:<br>▪ use everyday English words instead of complex words<br>▪ avoid meaningless terms<br>▪ use active voice and second person instead of third person<br>▪ use person-centered language, do not focus on circumstances, illness or disability<br>▪ write in an easily readable style<br>Sentences:<br>▪ use short and clear sentences<br>▪ avoid complex grammatical structures<br>▪ phrase positively rather than negatively | Text Length:<br>▪ adhere to convention<br>Form:<br>▪ break text up into paragraphs<br>Use of Tables/Visuals: - | Title:<br>▪ provide a good and relevant title<br>Content structure:<br>▪ order the text logically and let it flow naturally<br>▪ adhere to convention<br>▪ provide a first sentence that gives a concise introduction into text<br>Headlines: -<br>Content:<br>▪ provide answers to the questions: Who, What, Where, When, Why, How?<br>▪ give concrete everyday examples<br>▪ make sure risks are appropriately communicated (i.a.) | - | - | Purpose of text:<br>▪ to shortly report research to a general audience<br>Author of PLS:<br>▪ researchers<br>Review of PLS: -<br>Access:<br>▪ service providers may be responsible for the dissemination of research findings<br>Context: - |

| | | | | | | |
|---|---|---|---|---|---|---|
| **Dubé & Lapane (2014)**: Lay Abstracts and Summaries: Writing Advice for Scientists ("lay abstract" / "summaries"), multidisciplinary | Tone: -<br>Words:<br>▪ avoid long / multisyllabic / complicated words<br>▪ do not use acronyms (except commonly known ones)<br>▪ use active instead of passive voice<br>▪ check readability and reading level<br>Sentences:<br>▪ shorten sentences (but avoid choppy writing) | Text Length:<br>▪ depends on purpose<br>Form:<br>▪ check instructions<br>Use of Tables/Visuals: - | Title:<br>▪ state the main impact of your work for your audience with a simple phrase<br>Content structure:<br>▪ organize to make your story clear<br>▪ summarize the most important / relevant information at the beginning briefly<br>Headlines: -<br>Content: - | - | - | Purpose of text:<br>▪ the goal: a summary that is accessible to the public while true to science<br>Author of PLS: -<br>Review of PLS:<br>▪ review from target audience and other scientists of your field<br>Access: -<br>Context: - |
| **eLife (2017):** Plain-language summaries: How to write an eLife digest ("Plain Language Summaries" / "eLife digests"), life sciences | Tone:<br>-<br>Words:<br>▪ use words that are understandable to the widest group of readers<br>▪ avoid technical jargon (note that jargon can include common words that are used in a field-specific way<br>▪ if scientific terms must be used, define each at first use in more everyday-language<br>▪ only use well-known acronyms and do not use more than 3 acronyms overall<br>▪ rather use a few short words than one long one<br>▪ use verbs instead as nouns as much as possible | Text Length: -<br>Form: -<br>Use of Tables/Visuals: - | Title:<br>-<br>Content structure<br>▪ First sentence: Include something that most readers will be able to relate to<br>▪ Following sentences: Get gradually more specific<br>Headlines: -<br>Content:<br>▪ 4 questions that should be answered:<br>1. What background information would someone who is completely unfamiliar with your field need to know to understand the findings in your paper?<br>2. What exact research question did you set out to answer and why? | ▪ focus on findings highlighted in the title / abstract of your paper; explain them clearly and completely<br>▪ describe methodology with 1-2 sentences<br>▪ always mention which species, type of organism or cells you have studied | ▪ avoid hype / exaggeration | Purpose of text:<br>▪ explain findings of a eLife paper to a broader audience<br>Author of PLS:<br>▪ written by editors and writers working together with authors: the author of the scientific paper is asked to answer 4 questions; from that, the eLife Features team writes the digest<br>Review of PLS: -<br>Access: -<br>Context:<br>▪ published in a prominent position<br>▪ some are republished on social media platform<br>▪ encouragement to re-use PLS in other places |

| | | | | | | |
|---|---|---|---|---|---|---|
| | Sentences:<br>▪ active sentences<br>▪ all sentences should be shorter than 35 words | | 3. What are the most important findings of your paper? (always mention which species, type of organism or cells you have studied)<br>4. Who might eventually benefit from the findings of your study, and what would need to be done before we could achieve these benefits? | | | |
| **NIHR** (“Plain English Summaries”), health research | Tone:<br>▪ clearly and simply written<br>Words:<br>▪ avoid jargon, abbreviations and technical terms (provide explanation if you must use them)<br>▪ active rather than passive phrases<br>▪ link to glossary with lay-friendly terms<br>Sentences:<br>▪ keep short | Text Length:<br>▪ depends on funding programme<br>Form:<br>▪ break up the text, e.g. with bullet lists or headings<br>Use of Tables/Visuals: - | Title: -<br>Content structure:<br>▪ include, where appropriate, information on: Aim(s) of the research; Background of research; Design and methods used; Patient and public involvement; Dissemination of the findings<br>Headlines: -<br>Content: - | - | - | Purpose of text: -<br>Author of PLS:<br>▪ strong advice to involve patients, carers and/or members of the public<br>Review of PLS: -<br>Access: -<br>Context:<br>▪ links to resources are provided<br>▪ summary will be used on NIHR and other websites to describe the research |
| **American Geophysical Union** [several journals] (“Plain Language Summaries”), earth and space science | Tone:<br>▪ language and tone are different from scientific abstract<br>Words:<br>▪ avoid jargon, incl. undefined / excessive acronyms, field-specific terms, obscure / unnecessary long words, words that have different meaning to non-scientists<br>▪ use straight-forward descriptions<br>Sentences: - | Text Length: -<br>Form: -<br>Use of Tables/Visuals: - | Title: -<br>Content structure: -<br>Headlines: -<br>Content:<br>▪ develop your take home message by explaining what the research is about, what you found, and why it matters/what the impact is.<br>▪ provide enough context for those outside of your specific area of science will need more context<br>▪ highlight the novelty | - | - | Purpose of text:<br>▪ to explain your science to broader audiences<br>Author of PLS:<br>▪ authors of the scientific article<br>Review of PLS: -<br>Access: -<br>Context: - |

| | | | | | | |
|---|---|---|---|---|---|---|
| | | | and value of your research<br>▪ Think about: What was the research question (in the larger context of your field)? What did your study find? Why does it matter? What's the take-home message? | | | |
| **Halprin (2021)[1]:** Plain Language Summaries Explained in Plain Language, [several journals] ("Plain Language Summaries"), earth and space science | Tone: -<br>Words:<br>▪ *see above*<br>▪ define any field-specific terms<br>▪ explain acronyms if they have to be used<br>Sentences: - | Text Length:<br>▪ 200 words<br>Form: -<br>Use of Tables/Visuals: - | Title: -<br>Content structure: -<br>Headlines: -<br>Content:<br>▪ recommendation to use four elements (use 1-2 sentences each): 1) Topic Overview; 2) Paper Overview; 3) Findings Summary; 4) Key Takeaways | - | - | Purpose of text:<br>▪ *see above*<br>Author of PLS:<br>▪ *see above*<br>Review of PLS: -<br>Access: -<br>Context:<br>▪ examples are provided |
| **People and Nature** [journal] ("Plain Language Summaries"), human-ecology interactions | Tone:<br>▪ use clear and simple language<br>▪ simple and straightforward style<br>Words:<br>▪ avoid jargon<br>▪ avoid scientific terms; if you must use a scientific term, explain in clear and simple terms what it is<br>Sentences: - | Text Length:<br>▪ 250-300 words<br>Form: -<br>Use of Tables/Visuals:<br>▪ try and include a photo or image, anything that makes you work more accessible and interesting | Title:<br>▪ plain language title that says what the paper is about in clear terms (a bit like a newspaper headline)<br>▪ short (< 120 characters)<br>Content structure: -<br>Headlines: -<br>Content:<br>▪ include any important contextual background or findings that might make your work more relevant, interesting or memorable to the reader | - | - | Purpose of text:<br>▪ to raise the profile of your work<br>▪ to make it accessible to the widest possible audience<br>Author of PLS:<br>▪ author of paper<br>Review of PLS:<br>▪ upload PLS at revision stage with your work<br>▪ will be edited and returned to author if too much jargon<br>Access:<br>▪ freely available to read<br>Context:<br>▪ PLS will be added to journal blog, included with the paper<br>▪ encouraged to reuse PLS by posting it on own blog, share on social media |

| | | | | | | |
|---|---|---|---|---|---|---|
| **Autism** [journal] ("lay abstract"), all aspects of autism spectrum disorders and related developmental disabilities | Tone:<br>▪ easily understandable<br>Words:<br>▪ avoid technical terminology<br>Sentences: - | Text Length:<br>▪ max. 250 words<br>Form:<br>-<br>Use of Tables/Visuals: - | Title: -<br>Content structure: -<br>Headlines: -<br>Content:<br>▪ Consider the questions: What is already known about the topic? What does this paper add? Implications for practice, research and policy | ▪ avoid reporting of statistics | - | Purpose of text: -<br>Author of PLS:<br>▪ author of paper<br>Review of PLS:<br>▪ provide lay abstract as part of submission<br>Access:<br>▪ will be made widely available to general public and particularly to autistic people and their families<br>Context: - |
| **Taylor & Francis Group** [journal] ("Plain Language Summaries"), multidisciplinary | Tone:<br>▪ as objective as possible<br>▪ person-centered rather than focusing on the condition/illness<br>Words:<br>▪ free of jargon<br>▪ clear language<br>▪ easy-to-understand language instead of complex words<br>▪ active rather than passive voice<br>▪ avoid grammatical structures and abbreviations<br>▪ if you need to use a technical term, explain it the first time it is used<br>Sentences:<br>▪ short clear sentences<br>▪ phrase sentences in neutral language | Text Length:<br>▪ max. 250 words<br>Form:<br>▪ continuous prose format<br>Use of Tables/Visuals: - | Title: -<br>Content structure: -<br>Headlines: -<br>Content:<br>▪ Answer the questions: Why was the study done? What did the researchers do and find? What do the results mean? What is the objective influence on the wider field? | ▪ keep statements factual<br>▪ avoid providing opinions / speculation on study results and significance<br>▪ priority is that PLS is not misleading | - | Purpose of text:<br>▪ to communicate the significance of scientific research evidence to a broad evidence<br>Author of PLS:<br>▪ author of scientific article submitted to journal<br>Review of PLS:<br>▪ PLS is peer reviewed alongside the manuscript<br>Access:<br>▪ Open access license is the same as that of the published article<br>▪ freely available to read on website<br>Context:<br>▪ submit PLS as part of the manuscript or one year after (then incurring a fee)<br>▪ submission of PLS with scientific article is optional, but encouraged |

i.a. = if applicable; GRADE = Grading of Recommendations Assessment, Development and Evaluation (specific formal process for grading systematic reviews); SoF = Summary of Finding table (standardized form to report results of Cochrane Reviews)

[1] listed are only changes to the version mentioned above
