# Supplementary material for: Plain language summaries: A systematic review of theory, guidelines and empirical research
Source: PLoS One. 2022 Jun 6;17(6):e0268789. doi: 10.1371/journal.pone.0268789 (PMC9170105; doi:10.1371/journal.pone.0268789)
Supplement: S3 Table — (PDF) [file pone.0268789.s003.pdf]

S3 Table. Quantitative studies comparing PLSs with PLSs: criteria, outcomes and results.

**Study [sample]**	**criteria** *[characteristic category]*	**outcome** *[aim category]*	**results**
Santesso et al. (2015) [*n* = 143 members of the public and patients from 5 nations (Canada, Norway, Spain, Argentina, Italy)]	new format vs. current format:<br><br>- new format:<br>description of effects: qualitative and quantitative (absolute effects, natural frequencies in text; quantitative results in table)<br>quality of evidence: provided in table, according to GRADE<br>text structure: headings, question and answer format<br>content structure: flow of information according to linguistic framework principles<br><br>- current format:<br>description of effect: qualitative<br>quality of evidence: no criteria<br>text structure: paragraph of text<br>content structure: no criteria<br><br>*[Formal Attributes]*<br>*[Presentation of Results]*<br>*[Presentation of Quality of Evidence]*	Primary Outcome:<br>**A)** proportion of participants who correctly answered questions about the benefits and harms of the intervention and quality of evidence (multiple choice test, average of 5 questions)<br><br>Secondary Outcomes:<br>**B)** proportion of participants who correctly answered those 5 questions each<br>**C)** overall correct answers<br>**D)** comprehension of purpose of the summary<br>**E)** usability<br>**F)** ease of understanding<br>**G)** accessibility<br>**H)** preference for one format over the other<br><br>*[Knowledge]*<br>*[Understanding]*<br>*[Accessibility]*	**A)** proportion of participants who correctly answered comprehension questions on average was higher for the new format than the current (53% vs. 18%, $p < .001$)<br>**B)** proportion of participants who correctly answered single questions was higher for the new than the current format in 4 of 5 questions ($p < .001$)<br>**C)** more correctly answered questions by participants who read the new format compared to participants who read the current format (*Mdn* = 3, *IQR*: 1-4; vs. *Mdn* = 1, *IQR*: 0-1, $p < .001$)<br>**D)** no difference between formats regarding comprehension of the purpose of the study (32% vs. 45%, $p = .17$)<br>**E-G)** more participants who read the new format compared to the current format reported that the information was easy to find, reliable, that the summary presented most important effects and the presentation helped to make a decision (all $p < .05$), no stat. sign. difference regarding ease of understanding<br>**H)** greater preference for the new format (*p* unknown)
Silvagnoli et al. (2020) [*n* = 167, mostly higher-educated, from UK-based patient	readability low level of complexity (L1) vs. medium level of complexity (L2) vs. high level of complexity (L3)<br><br>*[Linguistic Characteristics]*	preference (score 1-4)<br><br>*[Accessibility]*	medium-level PLS with a readability that fits to an reading age of 14-17 years (L2) received highest preference ratings (PLS Psoriasis weighted means: 2.13 (L1) vs. 2.90 (L2) vs. 1.97 (L3); PLS Multiple Sclerosis weighted

association websites and Facebook patient support groups]			means: 2.37 (L1) vs. 2.47 (L2) vs. 2.40 (L3); PLS Rheumatoid Arthritis weightes means: 2.38 (L1) vs. 2.77 (L2) vs. 2.08 (L3), *p* unknown)
Alderdice et al. (2016) [n = 813 midwifery students from UK and Ireland]	conclusion vs. no conclusion<br><br>certain findings vs. uncertain findings<br><br>*[General Content]*	proportion of participants who identified the appropriate response to describe the main results of the review<br><br>*[Knowledge]*	no stat. sign. difference for conclusion vs. no conclusion for PLS with certain findings (63% vs. 61%; OR 1.13, 95% CI [0.41, 1.50]; RD 2.8%, 95% CI [-3.8, 9.5]; p = .41); stat. sign. more correct answers for conclusion vs. no conclusion for PLS with uncertain findings (45% vs. 37%; OR 1.35, 95% CI [1.02, 1.79]; RD 7.3%, 95% CI [0.60, 14.1]; p = .03)
Buljan et al. (2020, BMC Mes Red Methodol.), Trial 1 [n = 91, first-year medical students of University of Croatia]	positive framing (i.e., in terms of effectiveness)<br>vs. negative framing (i.e., in terms of ineffectiveness) of health evidence<br><br>*[Presentation of Results]*	Primary Outcomes:<br>**A)** perceived effectiveness of described treatment (score 3-30)<br>**B)** desire that treatment is offered by family doctor (score 3-30)<br>**C)** readiness to use the treatment (score 3-30)<br>Secondary Outcome:<br>**D)** comprehension (brief multiple choice knowledge test, score 0-12)<br><br>*[Knowledge]*<br>*[Empowerment]*	no differences for positive vs. negative framing:<br>**A)** M = 15.8 vs. M = 17.3;<br>*Mean Differenc*e = 1.53, 95% CI [-0.33, 3.39]; $BF_{10} = 1.31*10^{-4}$<br>**B)** M = 15.4 vs. M =16.1;<br>*Mean Differenc*e = 0.69 95% CI [-1.00, 2.37]; BF_{10} = .036<br>**C)** M = 16.6 vs. M =17.1;<br>*Mean Differenc*e = 0.52 95% CI [-1.39, 2.43]; BF_{10} = .035<br>**D)** M = 9.2 vs. M = 8.6;<br>*Mean Differenc*e = -0.51 95% CI [-1.43, 0.35]; $BF_{10} = 1.81*10^{-4}$
Buljan et al. (2020, BMC Mes Red Methodol.), Trial 2 [n = 245, students of University of Croatia and patients from hospitals or family practices]	presentation of treatment effectiveness as natural frequencies and side effects as percentages<br>vs. presentation of treatment effectiveness as percentages and side effects as natural frequencies<br><br>*[Presentation of Results]*	Primary Outcome:<br>**A)** comprehension (brief multiple choice knowledge test, score 0-2)<br>Secondary Outcomes:<br>**B)** preference for this type of format for health information (score 1-10)<br>**C)** perceived effectiveness of treatment for described medical condition (score 1-10)	**A)** "In Trial 2 we found no difference in CSR PLS comprehension when results were presented as natural frequencies or percentages (BF10 = 0.62, Bayesian t-test for independent samples)." (p.1)<br>**B), C)** "Our study showed no differences in readers' perceived effectiveness and readiness to use the described treatment [...] when the

		[Knowledge]<br>*[Accessibility]*	results were presented as frequencies vs percentages." (p.6)
Kirkpatrick et al. (2017)<br>[*n* = 60 members of the NETSCC's panel of public reviewers]	PLS written with 3 different strategies:<br><br>original PLS<br>vs. PLS rewritten with guideline by original author (author revised)<br>vs. PLS written with guideline by independent writer (edited)<br><br>[*Contextual Information*]	**A)** ease of understanding (score 1-4)<br>**B)** reading ease (Flesch reading ease score, 0-100)<br>**C)** free text comments<br><br>*[Understanding]*<br>*[Accessibility]*	**A)** no stat. sign. difference in terms of ease of understanding (original vs. edited, 56% vs. 72%; p = .06; author revised vs. original, 61% vs. 56%; p = .81; author revised vs. edited, 72% vs. 61%; p = .22)<br>**B)** both rewritten versions were significantly easier to read than the original PLS (p <.001); no stat. sign. difference between the rewritten versions (p = .12)<br>**C)** free text comments:<br>most frequent negative comments ($n \geq 20$) on jargon/terminology, title is not clear, insufficient detail, ambiguous language; most frequent positive comments ($n \geq 20$): general positive comments, headings useful (only revised and edited versions)<br>rather original than revised / edited PLS: need for headings, less detail
Kerwer et al. (2021)<br>[*n* = 166 German students]	PLS with subheadings<br>vs. without subheadings<br><br>*[Formal Attributes]*	**A)** perceived comprehensibility (score 1-8)<br>**B)** knowledge acquisition (proportion of correct responses)<br>**C)** perceived credibility (score 1-8)<br>**D)** perceived confidence in one's ability to evaluate the study (score 1-8)<br>**E)** perceived ability to make a decision without consulting an expert (score 1-8)<br><br>*[Understanding]*<br>*[Knowledge]*<br>*[Empowerment]*<br>*[Improvement of Research]*	**A)** higher comprehensibility for PLS with subheadings than without subheadings (M = 6.20, SD = 1.65 vs. M = 5.96, SD = 1.79, residual standard deviation = -.247, p < .001)<br>**B)** more correct responses for PLS with subheadings than without subheadings (78.68% vs. 75.91%; z = - 2.08, p = .019)<br>**C)** higher credibility for PLS with subheadings than without subheadings (M = 5.37, SD = 1.58 vs. M = 5.20, SD = 1.65, residual standard deviation = -.135, p < .05)<br>**D)** higher ability to evaluate for PLS with subheadings than without subheadings (M = 3.70, SD = 1.83 vs. M = 3.51, SD = 1.84, residual standard deviation = -.175, p < .01)

			**E)** higher ability to make a decision for PLS with subheadings than without subheadings (*M* = 3.10, *SD* = 1.76 vs. *M* = 2.78, *SD* = 1.71, residual standard deviation = -.267, $p < .001$)

PLS = Plain Language Summary; GRADE = Grading of Recommendations Assessment, Development, and Evaluation; Mdn = median; M = mean; IQR = interquartile range; OR = odds ratio; RD = risk difference; CI = confidence interval; CSR = Cochrane Systematic Review; BF = bayes factor; NETCSS = National Institute for Health Research Evaluation, Trials and Studies Coordinating Centre

Note. Only experimental conditions that investigate PLS criteria against each other are listed; further comparisons (e.g., with other summary formats) are not listed.
