# Supplementary material for: Plain language summaries: A systematic review of theory, guidelines and empirical research
Source: PLoS One. 2022 Jun 6;17(6):e0268789. doi: 10.1371/journal.pone.0268789 (PMC9170105; doi:10.1371/journal.pone.0268789)
Supplement: S1 File — (PDF) [file pone.0268789.s004.pdf]

S1 File. Search Terms.

Web of Science: last search on 2021-06-28; 4805 articles
TI = (“plain language summa*” OR “plain-language summa*” OR “lay summa*” OR “plain English summa*” OR “non-technical summa*” OR “non technical summa*” OR “nontechnical summa*” OR “summa* for layperson*” OR “systematic review* summa*” OR “evidence summa*” OR “lay abstract*” OR “plain language abstract*” OR “plain-language abstract*”OR “infographic*”) AND TI = (“quality” OR “standard*” OR “comparison” OR “effective” OR “evaluation” OR “critical review” OR “development” OR “user testing”) OR (AB = (“plain language summa*” OR “plain-language summa*” OR “lay summa*” OR “plain English summa*” OR “non-technical summa*” OR “non technical summa*” OR “nontechnical summa*” OR “summa* for layperson*” OR “systematic review* summa*” OR “evidence summa*” OR “lay abstract*” OR “plain language abstract*” OR “plain-language abstract*” OR “infographic*”) AND AB = (“quality” OR “standard*” OR “comparison” OR “effective” OR “evaluation” OR “critical review” OR “development” OR “user testing”))

PubMed: last search on 2021-06-28; 2495 articles
(“plain language summa*”[Title/Abstract] OR “plain-language summa*”[Title/Abstract] OR “lay summa*”[Title/Abstract] OR “plain English summa*”[Title/Abstract] OR “non-technical summa*”[Title/Abstract] OR “non technical summa*”[Title/Abstract] OR “nontechnical summa*”[Title/Abstract] OR “summa* for layperson*”[Title/Abstract] OR “systematic review* summa*”[Title/Abstract] OR “evidence summa*”[Title/Abstract] OR “non-technical summa*”[Title/Abstract] OR “lay abstract*”[Title/Abstract] OR “plain language abstract*”[Title/Abstract]OR “plain-language abstract*”[Title/Abstract] OR “infographic*”[Title/Abstract]) AND (“quality”[Title/Abstract] OR “standard*”[Title/Abstract] OR “comparison”[Title/Abstract] OR “effective”[Title/Abstract] OR “evaluation”[Title/Abstract] OR “critical review”[Title/Abstract] OR “development”[Title/Abstract] OR “user testing”[Title/Abstract])

PsycInfo: last search on 2021-07-02; 400 articles
3 ("plain language summa*" or "plain-language summa*" or "lay summa*" or "plain English summa*" or "non-technical summa*" or "non technical summa*" or "nontechnical summa*" or "summa* for layperson*" or "systematic review* summa*" or "evidence summa*" or "non-technical summa*" or "lay abstract*" or "plain language abstract*" or "plain-language abstract*" or "infographic*").ti. or ("plain language summa*" or "plain-language summa*" or "lay summa*" or "plain English summa*" or "non-technical summa*" or "non technical summa*" or "nontechnical summa*" or "summa* for layperson*" or "systematic review* summa*" or "evidence summa*" or "non-technical summa*" or "lay abstract*" or "plain language abstract*" or "plain-language abstract*" or "infographic*").ab. (550)
4 ("quality" or "standard*" or "comparison" or "effective" or "evaluation" or "critical review" or "development" or "user testing").ti. or ("quality" or "standard*" or "comparison" or "effective" or "evaluation" or "critical review" or "development" or "user testing").ab. (1387340)
5 3 and 4 (287)

PSYNDEX: Datum: last search on 2021-07-02; 14 articles
1 ("plain language summa*" or "plain-language summa*" or "lay summa*" or "plain English summa*" or "non-technical summa*" or "non technical summa*" or "nontechnical summa*" or "summa* for layperson*" or "systematic review* summa*" or "evidence summa*" or "non-technical summa*" or "lay abstract*" or "plain language abstract*" or "plain-language abstract*" or "infographic*").ti. or ("plain language summa*" or "plain-language summa*" or "lay summa*" or "plain English summa*" or "non-technical summa*" or "non technical summa*" or "nontechnical summa*" or "summa* for layperson*" or "systematic review* summa*" or "evidence summa*" or "non-technical summa*" or "lay abstract*" or "plain language abstract*" or "plain-language abstract*" or "infographic*").ab. (15)
2 ("quality" or "standard*" or "comparison" or "effective" or "evaluation" or "critical review" or "development" or "user testing").ti. or ("quality" or "standard*" or "comparison" or "effective" or "evaluation" or "critical review" or "development" or "user testing").ab. (75303)
3 1 and 2 (10)
