# Supplementary material for: Plain language summaries: A systematic review of theory, guidelines and empirical research
Source: PLoS One. 2022 Jun 6;17(6):e0268789. doi: 10.1371/journal.pone.0268789 (PMC9170105; doi:10.1371/journal.pone.0268789)
Supplement: S3 File — (PDF) [file pone.0268789.s006.pdf]

S3 File. Plain Language Summary about this Review.

Plain Language Summary

This text is a Plain Language Summary of our systematic review with the title "Plain Language Summaries: A Systematic Review of Theory, Guidelines, and Empirical Research" from 2021. The authors are Marlene Stoll, Martin Kewer, Klaus Lieb and Anita Chasiotis.

What was the aim of the review?

Background: Plain Language Summaries (PLS) are short texts that summarize a scientific study and that are written in a lay-friendly language. Because the concept is relatively new, there are different ideas of how PLS should be written and what the purpose of writing a PLS is.

Research questions: In this review, we had two aims.

1) We wanted to develop a PLS framework that describes the different components of PLS, the purpose of writing a PLS and how PLS can be studied. In order to do so, we brought together all theoretical ideas and guidelines about PLS,

2) We wanted to know how, according to current knowledge in research, how PLS should be written to communicate scientific studies to lay people in the best possible way. In order to do so, we reviewed scientific studies that investigate PLS.

Key message of this review

A lot of researchers have already thought theoretically about PLS, but there are not many comparable scientific studies on how to write PLS.

What was the way of working on this research question?

What kind of studies were searched? We searched for articles that studied PLS, discussed PLS, or that gave directions on how to write PLS.

Which studies were found? We found 90 articles. All 90 articles were used to investigate research question 1 about the theoretical ideas and guidelines regarding PLS. Ten of the 90 could be used to answer research question 2 about how PLS should be written.

What did we do with the studies? In all articles, we searched for information about what the components of PLS are ("PLS characteristics"), how a PLS should be written ("PLS criteria"), what the purpose of writing a PLS is ("PLS aims") and how we can measure if a PLS is good ("PLS outcome"). We gathered, structured and combined this information into a framework. Also, we summarized scientific studies and their results about how PLS should be written.

What are the most important results?

Results for Research Question 1:

Our framework describes:

- PLS consist of six main characteristics:
  1) Linguistic Features (e.g., which kind of words are used)
  2) Formal Features (e.g., how the PLS is structured, with or without bullet points)
  3) General Content (e.g., what the text narrates)
  4) How results are presented (e.g., how statistics are reported)
  5) How the quality of the method is presented (e.g., how the PLS reports bias risks)
  6) Context Features (e.g., who writes the PLS and where can you find it).

- The aims of PLS can be divided into six categories:
  1) PLS should provide access to the content of the study.
  2) PLS should be understandable.
  3) PLS should give the reader more knowledge.
  4) PLS should make possible that readers can use this knowledge, for example, when they are faced with challenges in their daily life.
  5) PLS should improve communication between researchers and the public.
  6) PLS should improve research as a whole.
- Around these characteristics and aims, a framework can be drawn that includes PLS criteria and outcomes.
- This framework provides people who want to develop a PLS guideline or who want to study PLS with a total overview of what can be considered when talking about or working on PLS.

Results for Research Question 2:
Since the 10 studies we used to answer research question 2 were very different from each other (e.g., some studies used very short and other studies used rather long texts as a PLS), conclusive results were difficult to identify. One reason is that the study authors had different fundamental ideas how a PLS should be written. However, there are insular clues that some PLS may be better than others due to certain features. One study showed that PLS should be written on a medium readability level (rather than a low or high level), another showed that PLS should be structured by headings. Another study showed that PLS should provide background information and report results not only with numbers but also with words that describe the results and another showed that PLS should be written in accordance with a guideline.
